# Activation of the pyrin inflammasome through the RhoA signaling pathway in FMF and HIDS

**DOI:** 10.1186/1546-0096-13-S1-O11

**Published:** 2015-09-28

**Authors:** YH Park, D Kastner, JJ Chae

**Affiliations:** 1 NHGRI, MCIDGB, Bethesda, USA

## Introduction

Mutations in the genes encoding pyrin and mevalonate kinase (MVK) cause the autoinflammatory diseases familial Mediterranean fever (FMF) and hyperimmunoglobulinemia D syndrome (HIDS), respectively. The inflammation of both diseases is mediated by interleukin-1β (IL-1β). Recently it has been reported that pyrin forms an inflammasome, a multiprotein complex that mediates the maturation of IL-1β by activating caspase-1, in response to bacterial modifications of RhoA. However, the precise molecular mechanism by which the pyrin inflammasome is activated is unknown.

## Objectives

To investigate the molecular mechanism of pyrin inflammasome activation and the molecular pathogenesis of FMF and HIDS.

## Methods

We studied IL-1β production in immune cells from wild type mice and from several knockout and knockin mouse strains, as well as from FMF and HIDS patients and healthy controls, in response to LPS and/or various other bacterial toxins, and in the presence of pharmacologic agents targeting the Rho GTPase or adenylate cyclase pathways. Protein interactions were studied by immunoprecipitation.

## Results

The Clostridial TcdB and C3 toxins, which inactivate RhoA, activate IL-1β maturation by a pathway that is *Mefv*-, *Asc-*, and *Caspase-1*-dependent, but *Nlrp3-, Nlrc4-,* and *Aim2-*independent. Leukocytes from FMF patients or FMF knockin mice produce IL-1β in response to LPS without a second signal; this is inhibited both by the bacterial CNF toxin, which activates RhoA, and by colchicine. In addition, the constitutive IL-1β secretion from FMF patients’ peripheral blood mononuclear cells (PBMCs) or macrophages of FMF-KI mice is potentiated by cAMP, which has a role in suppressing RhoA through PKA-mediated direct phosphorylation. RhoA inhibition-induced inflammasome activation is mediated by reduced activities of downstream RhoA-effector kinases, Rho-associated coiled-coil-containing protein kinase (ROCK) and protein kinase N1 (PKN1). Indeed, the kinase domain of the RhoA-effector kinases binds to pyrin directly and phosphorylates two serine residues (S208 and S242 of human pyrin). The phosphorylated pyrin is recognized by 14-3-3 proteins, which negatively regulate the pyrin inflammasome. The binding affinity of 14-3-3 proteins for the pyrin of FMF-KI mice is substantially lower than for wild-type mouse pyrin, which lacks a B30.2 orthologous domain. The constitutive IL-1β secretion from macrophages of FMF-KI mice as well as FMF or HIDS patients’ PBMCs is attenuated by activating RhoA-effector kinases. Defects in prenylation, seen in HIDS, lead to RhoA inactivation and consequent pyrin inflammasome activation.

## Conclusion

These data directly implicate Rho GTPase in the regulation of the pyrin inflammasome, and suggest that this pathway is also important in HIDS.

